# Designing Safer and Greener Antibiotics 

**DOI:** 10.3390/antibiotics2030419

**Published:** 2013-09-04

**Authors:** Andrew Jordan, Nicholas Gathergood

**Affiliations:** School of Chemical Sciences, Dublin City University, Glasnevin, Dublin 9, Ireland; E-Mail: andrew.jordan2@mail.dcu.ie

**Keywords:** antibiotic, resistance, environment, ionic liquid, green chemistry, biodegradation, toxicity

## Abstract

Since the production of the first pharmaceutically active molecules at the beginning of the 1900s, drug molecules and their metabolites have been observed in the environment in significant concentrations. In this review, the persistence of antibiotics in the environment and their associated effects on ecosystems, bacterial resistance and health effects will be examined. Solutions to these problems will also be discussed, including the pharmaceutical industries input, green chemistry, computer modeling and representative ionic liquid research.

## 1. Introduction

Environmental persistence of antibiotics has become a major concern for governments, regulation agencies and the pharmaceutical/cosmetic industries. Since industry began manufacturing chemicals on large scales in the early 20th century, some chemical compounds within waste, including antibiotics, have been observed in the environment, and in some cases without safe disposal [1]. Introduction into human, plant and wildlife populations has become inevitable and is now a major global issue [2]. Of great concern to the modern pharmaceutical industry is the continuing trend of microbial resistance to antibiotics. The current research ideology of creating more powerful and broad spectrum treatments, and more resilient compounds to tackle the threat of microbial resistance (through methods of combinatorial synthesis, high throughput screening and rational drug design), could be a double edged sword. On increasing the use of more robust and broad spectrum antibiotics comes a potential increase in their concentration in the environment due to a decrease in their biodegradability. To the best of the authors’ knowledge, there is currently no drive towards creating biodegradable antibiotics. The Golden Age of antibiotics has passed, with decreasing numbers of successful antibiotics being produced every decade. Research institutions and the pharmaceutical industry must adapt and overcome the ongoing problems, issues and challenges encountered in the 21st century [3].

The search for greener and safer antimicrobial drugs, which have a reduced impact on the environment can be determined by three main factors:

(1) The effect on the environment due to the synthetic route selected (number of steps, toxicity of reagents, and treatment of waste). *i.e.*, a shorter, cleaner and greener synthesis is preferred.
(2) Inclusion of impact on the environment studies and lifecycle assessment as part of the drug development process. This should preferably avoid the selection of compounds expected to have a major adverse effect on the environment.
(3) A comprehensive evaluation of the toxicity/biodegradation and possible persistence problem of the antibiotic in the environment. This review will focus on the second factor, with the first and third points included for context rather than a review.

## 2. Antibiotics

Pharmaceuticals are especially designed to interact with biological systems, so their introduction into the environment may lead to an unintended impact in nature (as long as concentrations reach significant levels). For example, it has long been known that excessive amounts of hormones, especially estradiol from birth control pharmaceuticals, can drastically interfere with aquatic species immune systems. Estrogen and androgens can interfere with fish endocrine system and cause disruption to reproductive cycles [4]. These observations of pharmaceutical interference with animal and bacterial lifecycles has raised the question about whether compounds previously thought to be benign are having a damaging effect to the environment.

## 3. Penicillin: The First Drug Casualty to Bacterial Resistance

**Figure 1:**
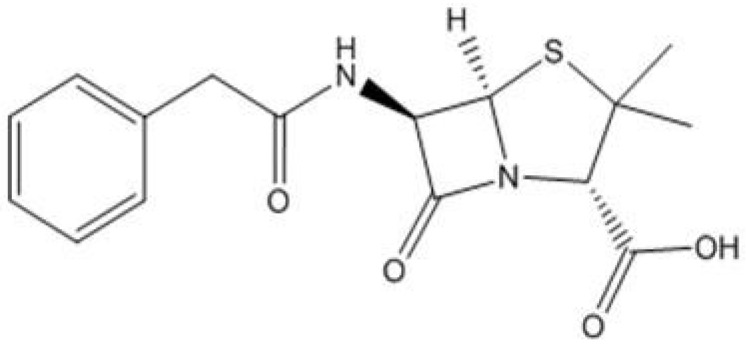
Penicillin G, the gold standard Penicillin antibiotic.

There are a plethora of antibiotics available for human and animal use with many classified into the following categories: β-Lactam/Penicillin, Cephalosporins, Carbapenems, Aminoglycoside, Quinolone, Macrolide, Sulphonamides, Tetracyclines and Peptide antibiotics. There are major points of difference, as well as similarities, between the categories and their modes of actions and administrations. Penicillin, the first antibiotic, discovered by Sir Alexander Flemming in 1928, has widespread use and has undergone many structural changes at the hands of synthetic chemists to give an extensive library of derivatives. Due to an over reliance and ubiquitous use of Penicillin G, Figure 1, during the 1950s, *S. aureus* evolved and developed a resistance to treatments leading to increasing amounts of penicillin resistant *S. aureus* infections found in hospitals. *Methicillin Resistant Staphylococcus Aureus* (MRSA) was first reported in 1961, with a critical level of infections reached in the mid to late 1960s. Due to this resistance, MRSA strains are currently one of the most serious pathogens in hospital and community healthcare locations. Examples of antibiotic resistance that originated from healthcare overuse of a drug were reported by Lanzky and Smyth [5,6].

## 4. Resistance in the Environment

Concentrations of pharmaceuticals in the environment is the major point of contention between proponents of the theory that any level of antibiotics in the environment will promote resistance and those who believe only elevated concentrations can lead to a noticeable effect on bacteria promoting resistance genes [7,8]. It has been shown that there is a natural background level of a drug resistant bacteria species that have a non-transmissible effect in the environment [9]. There are also comparable amounts of resistant bacteria that can cause microbial infections in humans, and these species are of particular concern, however, they appear to be very local in their distribution. In the aforementioned study, it was shown that present in dairy farms soil and canal water (near the same dairy farm) were large amounts of resistant bacteria, including Vancomycin and Tetracycline resistant types. The direct affect of antibiotic usage in animal feed was then shown to have affected a canal run off from the dairy farm at the same location. A decrease of one order of magnitude was observed from direct soil and manure samples compared to water samples in the vicinity. Of note is a further decrease in the order of magnitude when samples from a local park waterway were analyzed. This shows, in this case, that bacterial resistance is location specific, see Table 1 for type of sites analyzed.

**Table 1:** Total bacterial counts of sites studied by Esiobu *et al*. Adapted with permission from [9].

*Sampling Site*			*Log (CFU/g or 100 mL)*				
	*Total counts*	*Gram-negative*	*Gram-positive*	*Pseudomonads*	*E. coli*	*Enterobacter*	*Enterococci*
Dairy farm soil	8.15	8.02	7.54	5.18	2.47	4.8	3.08
Dairy farm cow manure	6.6	5.62	6.55	2.88	2.5	3.45	1.22
Dairy canal water	5.48	5.31	4.99	2.2	1.75	2.01	1.1
Residential garden soil	6.3	5.9	6.08	3.92	2.4	3.55	2.35
Lake by hospital	5.9	5.77	5.32	3.5	2.35	2.88	1.99
Public park canal water	4.7	4.6	4.05	1.8	0.8	1.27	2.01
Residential estate lake	6.51	6.48	5.32	2.1	2.02	3.5	1.04

Similarly in a study conducted by Herwig, R.P. *et al*., [10] it was shown that background resistance levels of bacteria to Oxytetracycline (OTC), Amoxicillin and Romet 30 (a combination drug comprised of Sulfadimethoxine and Ormetoprim) were around 5% levels. All the aforementioned drugs are used by fish farms in Puget Sound, WA, USA. When compared to levels directly below three fish farms in the area the level or resistance rapidly increased for OTC and Romet (but not for Amoxicillin). On one of the farms a >20% increase was noted, while for the other two farms a ~5%–10% increase was observed. The farm that showed the greatest increase had also used the largest quantities of antibiotics.

**Figure 2:**
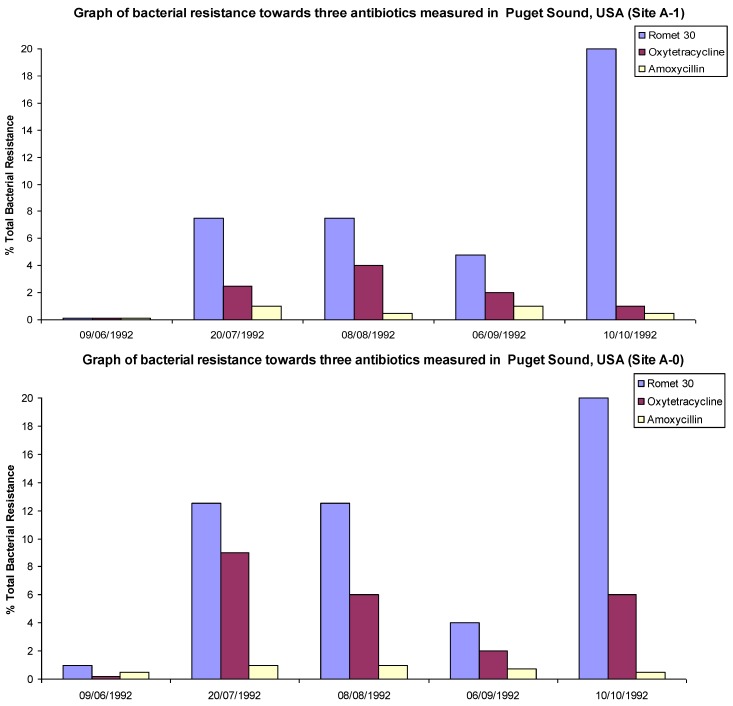
Graphs of % total resistance of bacteria trended with total drug sediment at a fish farm in Puget Sound, WA, USA. Adapted with permission from [10].

The increase in resistant bacterial strains occurred at the same time that large quantities of antibiotics were introduced to the fish populations. The resistant populations then stabilized and subsided slightly over the course of the study as selective pressure from antibiotics had been removed from the environment, see Figure 2. It has been suggested that the lack of increase in Amoxicillin resistance in the local environment was due to either not enough time being allowed to observe an increase in resistance, or possibly due to rapid degradation of amoxicillin in the environment avoiding accumulation in the sea floor sediments [11]. The suggested mechanism of this degradation is the opening of the lactam ring structure and not the hydrolysis of the amide bond [12].

A study conducted by Schnabel and Jones in 1999 [8] in orchards in the US illustrated that selective pressure from using tetracyclines and streptomycin on fruit, to control fire blight amongst other infections, promoted development of resistant bacteria. There were two orchards involved in the study, one of which had a history of heavy usage of tetracycline. The highest numbers of tetracycline resistant bacteria were found in the orchard with the heaviest usage of tetracycline, even on trees that had no history of direct exposure to tetracycline usage in the vicinity [8].

The evidence from studies conducted in localities where a good control and large sample area are used cannot be ignored, especially when such diverse locations suggest the same results. What is clear from such studies is that introducing antibacterial and antibiotic compounds to an environment increases the numbers of resistant bacteria detectable, and can occur rapidly. As distance increases from the area sampled, the opposite occurs to the number of resistant strains detectable [10]. The major concern highlighted by these studies is that there is the possibility of both horizontal (and vertical) transfer of resistance elements from bacterial species co-habiting an environment [7,13]. While bacterial resistance in fish populations may not appear to be of significant to human health, it is of major concern if resistance to antibiotics (e.g., tetracycline) transfers to a bacteria pathogenic to humans, especially if the bacteria is then reintroduced to humans by consuming the fish product.

Research conducted by Olsen and coworkers in 1999 [14] and Harbottle and coworkers in 2006 [7] demonstrated that plasmids, transposons and integrons are among the elements that promote rapid genetic resistance transfer between bacteria. Bacteria expressing resistance genes can reach an aquatic environment and horizontally transfer resistant genes to native bacteria creating a pool of genetic resistance that remains in the environment [7,14]

*“A variety of antibiotics and their metabolites at sub-inhibitory level concentrations are suspected to expand resistance genes in the environment. However, knowledge is limited on the causal correlation of trace antibiotics or their metabolites with resistance proliferation”*.

[15]

The work conducted by Caian Fan and coworkers in 2011 [15] demonstrated that at sub-inhibitory concentrations of erythromycin (as low as 50 μg/L) can provide sufficient pressure to induce bacterial resistance. These levels correspond with concentrations as low as found in the environment [15]. Further work is required conducted at these trace concentration levels to determine the overall effect of antibiotics in the environment on permanently influencing bacterial resistance.

## 5. Damage to Ecosystems

Proliferating bacterial resistance is only one area of concern regarding antibiotics present in the environment. Since antibiotics are designed to inhibit bacterial growth, they also have the potential to disrupt ecosystems and impair ecological functions. 

The presence of antibiotics in soil has also been shown to impair nitrification/denitrification processes. It was demonstrated by Kotzerke *et al.* 2008 [16] that introducing sulfadiazine to manure significantly reduced the growth enhancing effects of manure on ammonium oxidizing bacteria. The reduced ability of ammonium oxidizing bacteria was offset by an increase in ammonium oxidising archae [16]. Similar effects of depressed denitrification processes were observed by Costanzo and coworkers in 2005 [17].

The presence of antibiotics in aquatic systems has also been demonstrated to have an effect on algae. In a study conducted by Lanzky and coworkers in 1997 [5], it was shown that Metronidazole—a drug used to treat, amongst other things, amebic and protozoic infections—affected algal populations. As it is such an important food source, any change in the algal population densities directly affects other creatures in the same ecosystem [5].

Similarly, it has been demonstrated that the commonly used Chloramphenicol and related phenicol compounds can directly inhibit algal growth [18]. In another study, the concentrations of antibiotics required to inhibit algal growth were much lower than the phenicol study and went as low as 0.004 mg/L concentrations of amoxicillin required to inhibit *M. aeruginosa* growth [19].

## 6. Drug Development—Benign by Design

There is a constant battle for survival being fought between the medicinal chemist and bacteria—the goal to be one step ahead of bacterial resistance and provide drugs that are safe for human consumption. Pharmaceutical companies are also continually striving to produce more effective drugs, improve the quality of human life, and satisfy their shareholders. Due to the intense pressures involved in drug design processes, and lack of incentives, there are currently no prerequisites that the drugs on the market need be readily broken down in the environment. Low level concentrations of antibiotics are being allowed to persist in the environment, exposed to bacterial populations. The fates of pharmaceuticals in the environment are well documented and the evidence in the previous section points towards potential long term detrimental effects.

Taking into account the potentially damaging effects of pharmaceuticals on the environment, and the proliferation of bacterial resistance, would it not be better to build in mechanisms by which antibiotics can readily degrade once introduced into the environment? The concept of benign by design is not new and means that ease of degradation is taken into account before an active pharmaceutical ingredient is even envisaged and synthesized. By adding design criteria of ready biodegradability into a drug discovery project, we propose that any lead compounds from such a discovery process are less likely to cause environmental persistence. Currently, however, there are no incentives for pharmaceutical companies to include such criteria in their drug discovery programs. Until there are incentives provided to include lifecycle engineering of drugs as a regulation criteria then pharmaceutical companies will continue to produced APIs with broad spectra of biodegradation profiles [20]. It must also be recognised that the addition of extra hurdles for a lead compound to overcome before reaching market further increases the burden on the pharmaceutical industry. If development of greener and safer antibiotics can be directly linked to an economic (as well as environmental) benefit, this will promote research in this area.

## 7. Green Chemistry Principles

A set of guidelines known as the “12 Principles of Green Chemistry” have directed green chemists in their research endeavors since the late 1990s, and were proposed by Anastas and Warner. The principles provide a framework that when adhered to can lead to synthetically useful molecules that are conceived, used, and disposed of, in a more environmentally friendly manner [21]. The principles defined are as follows:

1. Prevention
  It is better to prevent waste than treating waste after it is produced [22];
2. Atom Economy
  Synthetic methods should be employed so as to maximize incorporation of all reagents used, in the final product [23];
3. Less Hazardous Chemical Syntheses
  When possible reagents and synthetic methods less toxic to human health and the environment should be used [24];
4. Designing Safer Chemicals
  Chemicals should be designed, fit for purpose, with minimum toxicity to humans and the environment [25];
5. Safer Solvents and Auxiliaries
  The use of auxiliary substances such as solvents, drying agents etc. should be reduced when possible [26];
6. Design for Energy Efficiency
  The energy required to perform a chemical process should be kept to a minimum, e.g., temperature and pressure conditions of reactions [27];
7. Use of Renewable Feedstocks
  Raw materials should be from a renewable source if possible [28];
8. Reduce Derivatives
  Unnecessary use of protecting groups and structural modifications should be avoided to minimise waste production and energy consumption [21,29].
9. Catalysis
  Employing catalysts reduces waste and energy requirements and should be used when possible and appropriate, *i.e.*, selectivity [30].
10. Design for Degradation
  Synthetic molecules should be designed to breakdown in the environment after use to avoid chronic build-up effects. Eventual fate in the environment must be considered [31].
11. Real-time Analysis for Pollution Prevention
  Real-time process monitoring to control and prevent the production of potentially hazardous materials should be employed [32].
12. Inherently Safer Chemistry for Accident Prevention
  Safer reagents, procedures and processes should be employed to reduce the change of accidents and exposure of chemicals to the environment and people [33].

By incorporating the design principles outlined above, chemists from academia and industry can effectively produce molecules that are fit for purpose and pose far less of a risk to the environment. By including biodegradation, toxicity, sustainability and life cycle assessment in design criteria, ranges of safer compounds can be effectively produced with reduced impact on ecosystems.

## 8. Green Research Processes

To analyze a pharmaceutical process or a research project one must first produce a set of standards, tests or a scale with which to measure the projects “greenness”. Pioneers of green chemistry metrics in the early 1990s established concepts such as “Atom Economy” (Trost) [23], “E-factor” (Sheldon) [22], “Andraos Reaction Mass Efficiency” [34], “GSK Reaction Mass Efficiency”, “Process Mass Intensity”, “Solvent Intensity” [35,36,37]. The concept is that every chemical process can be analyzed and its greenness determined, compared and improved.

In recent years, there has been a drive in the pharmaceutical industry to produce an API with a minimum amount of waste. There is often an economic incentive as well as benefit to the environment. The largest materials usage in the pharmaceutical industry is solvents, which make up to 90% of material usage producing bulk API [38]. This makes solvent choice of paramount importance. Pharmaceutical companies have reported solvent selection guides, which illustrate pros and cons of common solvents used in their processes, including common chlorinated solvents and their alternatives [29].

The atom economy of a reaction represents the percentage incorporation of starting materials into the final product, so a reaction with 100% atom economy will have complete incorporation of all starting materials into the product. For example, a Diels-Alder reaction can have up to 100% atom economy, whereas a Wittig reaction in general is rated with a very low value. However, percentage yield is not a consideration when calculating the atom economy. In addition, atom economy, which can be very easily calculated from a reaction scheme, does not take into account solvent and bulk materials used in the process (e.g., product isolation). The Sheldon “Environmental Factor”, or E-factor, does include solvents and waste parameters and is a simple calculation of waste quantity divided by product quantity; the lower the number the more environmentally acceptable the process. 

## 9. Life Cycle Assessment

Life cycle assessment (LCA) is the study of a chemical compounds affects the environment, from inception to destruction. LCA is now recognized as a distinct field of study and can potentially analyze the overall impact of any step of a process to the environment. There are currently two ISO standards in place regarding Life Cycle Assessment. They are ISO 14044 and ISO 14040. These international standards outline a framework for LCA by breaking any assessment down into four phases. These phases are:

(1) Goal, Scope and Definition of a Study;
(2) The Life Cycle Inventory—A Comprehensive and Exhaustive Analysis of all Interactions of a Product with the Environment;
(3) Life Cycle Impact Assessment—Analyzing the Data Gathered in Phase 2 to Assess the Overall Impact of the Object of the Study;
(4) Interpretation of Results—Interpreting Consequences of Data Gathered from Phase 2 and 3, Making an Informed Conclusion and Proposing Suggested Courses of Action.

In a 2004 case study performed by GSK, in association with North Carolina State University, a “cradle to gate” of API production was carried out. The study fully assessed every material on a particular production tree, analyzed energy uses in all steps, and included waste streams and transport energy in the considerations. Using GSK’s own in-house metrics the impacts of API production were fully analyzed from when the raw materials entered production to leaving the gate (Note: The fate of these products downstream was not included.). The results of the study found that solvent use is accountable for 75% of total energy use in the API production, and that great benefits can be obtained by more efficient solvent disposal, recycling and optimization [39].

## 10. ADMET—Absorption, Distribution, Metabolism, Excretion, Toxicity

ADME is one of the oldest acronyms adopted by medicinal chemists and is a broad description of a drugs lifecycle in the human body. The shortcomings with such a coverall are that once the drug is excreted its effects are no longer considered. A more recent appendage to ADME is “Toxicity” to the acronym. This highlights the importance of toxicity parameters as part of the drug development process, but is usually limited to effects within the body (*i.e.*, not post-excretion). Enzymes can critically activate or deactivate a parent drug molecule into harmless metabolites or biologically active compounds, and it is the latter that are of concern. A drive to understand the toxicity of potential metabolites has emerged using *in silico* methods (QSAR *etc*.). *In silico* modeling and predictive modeling can be employed during the drug development phase, along with traditional screening assays, to determine to within an acceptable degree of confidence, a drugs biological activity profile. The only shortcomings are datasets available to base these predictive calculations. This will improve over time making *in silico* screening more attractive and thus a reliable resource to complement traditional screening methods [40]. Other parameters beyond toxicity of a drug candidate can also be predicted including physiochemical properties (Log P, critical micelle concentration *etc*.), and can be useful datapoints in determining a drug’s behavior inside the body and once released into the environment [41,42]. Furthermore, forays to predict biodegradation have been made using software suites such as META [43], BIOWIN [44], CATABOL [45] and TOPKAT [46].

Computational screening methodologies are usually preliminary studies before Phase 0 or Pre-Phase I clinical trials. Being in a position to analyze a drugs performance without increasing dosage to critical levels is a powerful asset. The ideology behind Phase 0 trials is that a non therapeutic quantity of the pharmaceutically active compound is administered to a trial participant. The dose while non-therapeutic can still have a measurable pharmacological activity in the body. This sub therapeutic level allows for monitoring of ADMET properties of potential drug candidates with minimal risk to trial participants health [47,48]. The results from Phase 0 trials can then be fed back into the drug design program and allow for alterations to a drug candidate to be made as early as possible in a drugs development lifecycle. The benefits of early screening are both financial and environmental, as minimum risk to clinical trial participants occurs, and a wealth of information can be gathered regarding a drug molecules toxicity before phase I trials [49].

With simple green chemistry metrics (*vida supra*), one can begin to evaluate the environmental impact of research and production of pharmaceuticals. For example, the footprint of antibiotic production can be determined by the E-factors and waste emissions. However, methods of data analysis always have limitations, which must be considered when interpreting the results. A case in point being that E-factor does not take into account the toxicity of the waste streams being produced. A process may be green according to Atom Economy and also have a small environmental factor according to E-factor but the toxicity of the waste is not considered. Toxicity of waste should be taken into account as part of an informed drug design program. Increasing the quality of datasets that *in silico* screening relies on will also improve the accuracy and reliability of such methods, enabling rational design of greener drugs. By including environmental toxicity into a drug design program is by far one of the most important steps towards a greener pharmaceutical industry can be envisaged.

## 11. Green Chemistry—Pharmaceutical Successes

Within the pharmaceutical industry, there have been a number of green chemistry success stories. The annual ACS Presidential Green Chemistry Challenge awards highlight numerous areas of green chemistry research especially industrial processes, which have been made more efficient and environmentally friendly. The areas of research promoted include polymer chemistry, antivirals, pesticides, catalytic transformations, enzymatic transformations and renewable feedstock research.

The synthesis of Sitaglitpin, an API used to treat type 2 diabetes, is a recent and prominent example of what can be achieved by industrial collaboration and coordinated efforts with green chemistry research. An incredible improvement was achieved by Merck and Codexis. The greener synthesis once optimized:

*“...eliminates the high-pressure hydrogenation, all metals (rhodium and iron), and the wasteful chiral purification step. The benefits of the new process include a 56 percent improvement in productivity with the existing equipment, a 10–13 percent overall increase in yield, and a 19 percent reduction in overall waste generation”*.

[50]

Similarly the replacement of Baccattin III in the synthesis of Taxol—Baccattin III formation being a major breakthrough by reducing the synthesis of the API from 40 steps to 11 transformations—using plant cell fermentation technology allows BMS to

*“...improve the sustainability of the paclitaxel supply, allows year-round harvest, and eliminates solid biomass waste. Compared to the semisynthesis from 10-DAB, the PCF process has no chemical transformations, thereby eliminating six intermediates. During its first five years, the PCF process will eliminate an estimated 71,000 pounds of hazardous chemicals and other materials. In addition, the PCF process eliminates 10 solvents and 6 drying steps, saving a considerable amount of energy. BMS is now manufacturing paclitaxel using only plant cell cultures”*.

[51]

The above examples bring together engineering, biology and chemistry to solve problems, which lead to more environmentally sustainable and financially viable processes. However, though there have been some exceptional successes within the pharmaceutical industry, there is a notable observation that no awards have yet been presented for improved antibiotic manufacture. 

## 12. Ionic Liquids (ILs) as Green API’s

The research areas of interest to the Gathergood research group include ILs surfactant molecules, solvents and organocatalysts. Projects conducted by the group incorporate biodegradation, ecotoxicity and antimicrobial activity studies. By including these parameters into the research conducted, the potential environmental impact of developing such molecules can be analyzed and data sets on novel molecules can be produced. The data collected assists further understanding of how biodegradation and toxicity of new molecules can be predicted, and ultimately direct and promote research at the early stage of the development cycle.

The area of IL research offers exciting new possibilities in safer and greener medicinal products. There has been an upsurge in the past two decades into research being conducted in the field of ILs. ILs have been described as molten salts that are entirely ionic in nature, comprising both a cationic and anionic species and by definition having a melting point below 100 °C [52]. The physical, chemical and biological properties of these salts can be “tuned” for a particular function. These reaction media have been designed as replacements for conventionally used organic solvents, and research has been widely reported on their applications in the area of “green” chemistry [52,53,54]

Although ILs were first synthesized over one hundred years ago, the term ionic liquid is relatively modern. Previously the compounds synthesized would have been classed under a variety of terms including—molten salts, surfactants, detergents and electrolytes. The last term, electrolytes, has particular significance to the design of biodegradable ionic liquids. Electrolytes need to be stable, usually under aggressive conditions e.g., in a battery, capacitor or solar cell, and research in this area was targeted towards robust structures with long shelf lives. Of interest since the 1940s, the design of chemically inert (persistent, non-biodegradable) electrolytes was proceeding concurrently with the search for biodegradable surfactants! In addition, high antimicrobial toxicity of quaternary ammonium compounds (QAC’s) comprised of an ionic head group and long alkyl of PEG chain is widely reported. This can also have a significant negative impact on the environment especially if biodegradation is poor, leading to bioaccumulation. 

**Figure 3:**
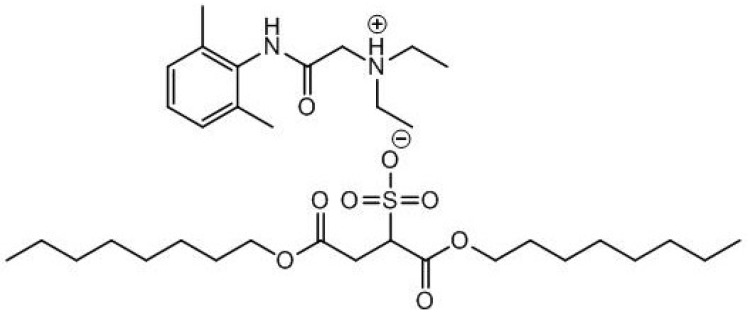
Lidocaine docusate IL.

The toxicity of some ILs is undesirable in most “green” chemistry applications, but this tuneable property may be exploited and used beneficially in the development of antimicrobials, pharmaceuticals, solvents and surfactants. A number of groups have recently demonstrated the potential biological applications of ionic liquids in drug delivery and as active pharmaceutical ingredients (APIs). Rogers and co-workers prepared a room temperature IL based on the local anaesthetic Lidocaine [55]. The IL (see Figure 3), was prepared by exchanging the anion with docusate. The IL analogue of the drug delivered longer pain relief and a slow-release mechanism of drug delivery. Antimicrobial ILs have also been applied as additives for medical devices. Dual functional ILs have been reported with plasticizing effects on poly(vinyl chloride) (widely used in the production of medical devices) along with antibacterial activities. Contamination of medical devices with resistant bacteria biofilms, could be treated with these antibacterial ILs [56].

Antibiofilm properties of ILs have been reported in a number of papers (including recently by Gilmore *et al.*, 2009) [57]. Alterations can be envisaged by cation/anionic group substitutions or the additions of various side chains. The potential for diversity using an IL as a backbone for building a therapeutic is vast [57].

Incorporation of ILs with antimicrobial activity as fillers for bone cement and dental applications has been demonstrated by Marr and coworkers in 2009 [58]. Ionogels containing an IL provide an effective support for catalytic reactions, boasting excellent recyclability [58]. We postulate that similar supports could be employed for biologically active ILs. This in turn could lead to a drug delivery option or site specific application. Tailoring a silica support could facilitate either the slow release of the therapeutic agent or the immobilization of the IL by entrapment and protecting the IL. This would have the potential to allow the pharmaceutical/antimicrobial action of the IL to be controlled, acting only in the desired region of the body. This would be especially advantageous in implants where longevity due to site infection is an issue [59].

## 13. Toxicity

Within the Gathergood group, the design criteria for compounds synthesized includes biodegradation, ecotoxicity, and antimicrobial activity studies. Preliminary biological screening, toxicology screening and biodegradation screening was conducted on compounds synthesized. Readily biodegradation studies are performed using the Closed Bottle Test OECD 301D and CO_2_ Head Space Test ISO 14593. 

**Figure 4:**
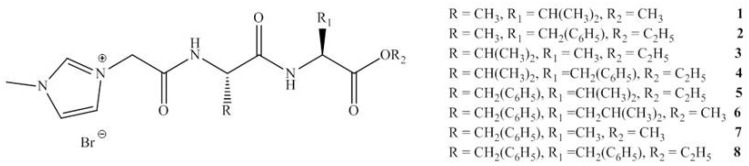
General structure of dipeptide ILs synthesized.

**Figure 5:**
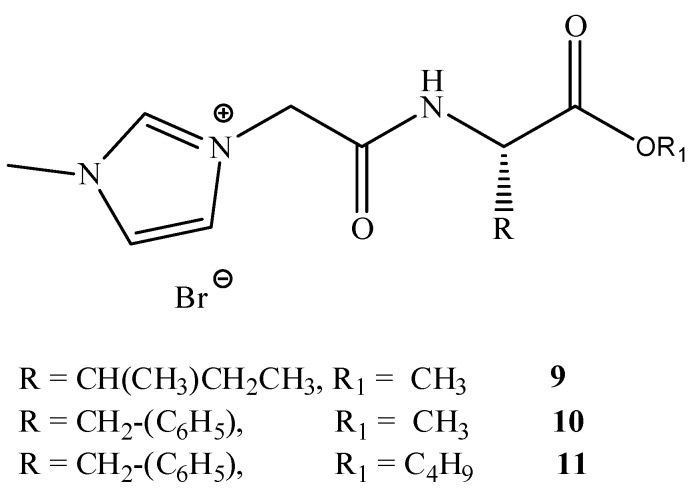
General structures of amino acid derived ILs.

This group reported in 2012 an investigation studying the incorporation of amino acids into IL cations. A series of amino acid ILs (**1**–**11)**, including the dipeptide ILs (**1**–**8)**, were produced (see Figure 4, Figure 5) which lead to the pharmaceutically significant molecule (**5**). *In vitro* antibacterial and antifungal activities of these ILs were investigated against a range of bacteria and fungi, including clinically resistant strains with MIC values reported (see Figure 4 and Table 2). 

**Table 2:** MIC (μM, IC_95_) values obtained for dipeptidyl ILs **5**–**8**. **1**–**4** exhibited MIC values > 2 mM and are not included in the table.

Organism	Time (h)	IL			
		5	6	7	8
*S. aureus* (ATCC 6538)	24, 48	**500**, **1000**	>2000, >2000	>2000, >2000	**1000**, **1000**
*Methicillin-res.S.a.* (HK5996/08)	24, 48	**125**, **500**	>2000, >2000	>2000, >2000	**2000**, **2000**
*S. epidermidis* (HK6966/08)	24, 48	**500**, >2000	**2000**, >2000	**1000**, >2000	**2000**, >2000
*Enterococcus sp.* (HK14365/08)	24, 48	**2000**, >2000	>2000, >2000	>2000, >2000	**2000**, **2000**
*E. coli* (ATCC 8739)	24, 48	>2000, >2000	>2000, >2000	>2000, >2000	>2000, >2000
*K. pneumoniae* (HK11750/08)	24, 48	>2000, >2000	>2000, >2000	>2000, >2000	>2000, >2000
*K. pneumoniae-ESBL* (HK14368/08)	24, 48	>2000, >2000	>2000, >2000	>2000, >2000	>2000, >2000
*P. aeruginosa* (ATCC 9027)	24, 48	>2000, >2000	>2000, >2000	>2000, >2000	>2000, >2000

## 14. Biodegradation

To date, biodegradable surfactants have been developed. The dominant design strategy adopted to promote biodegradation has been the inclusion of ether and ester linkages, however examples of readily biodegradable amide bond containing amino acids, including gemini surfactants, have been synthesized [60]. To the compound of interest is appended an ester or ether side chain, see Table 3, which has been shown to increase biodegradability through new enzymatic hydrolysis steps. A drawback to this approach is the rapid hydrolysis of ester groups under a wide range of conditions (acid, base, thermal, *etc*.). An innovative approach being studied by the Gathergood group is the additional introduction of amide groups via amino acids into ILs, see Figure 5. This has subsequently led IL researchers to assess the various biological properties, namely toxicity, biostability and biodegradability (see Table 4, Table 5), of these “green” chemicals [52,53,61,62,63]

**Table 3:** Examples of some oxygen functionalized ILs with octyl sulphate anions (OctOSO_3_^−^) (**12**–**14**) and their biodegradation results. For comparison, an amide functionalized ILs (**15**) is included. Note: Poor biodegradability of first generation of amide ILs. **SDS** = Sodium dodecyl sulfate.

CO_2_ Headspace Test	% Biodegradation				
Compound	0 day	7 day	15 day	21 day	28 day
**SDS**	0	81	85	90	92
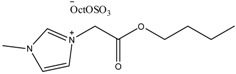 **12**	0	45	54	56	59
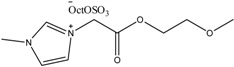 **13**	0	54	59	59	59
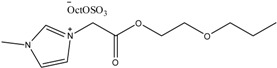 **14**	0	51	58	61	65
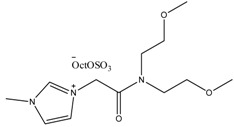 **15**	0	26	30	29	29

**Table 4:** Biodegradation data for amino acid derived ILs (**6**) and **SDS** internal standard. (**6**) is classed as readily biodegradable (ISO 14593).

CO_2_ Headspace Test	% Biodegradation				
Compound	0 day	6 day	13 day	20 day	28 day
**SDS**	0	67	91	91	87
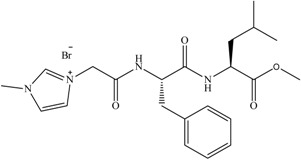 **6**	0	42	65	68	64

**Table 5:** Biodegradation data for amino acid derived ILs (**11**) and **SDS** internal standard. (**11**) is classed as readily biodegradable (ISO 14593).

CO_2_ Headspace Test	% Biodegradation				
Compound	0 day	7 day	15 day	21 day	28 day
**SDS**	0	78	89	91	94
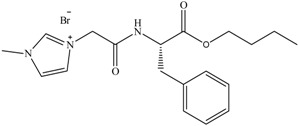 **11**	0	16	59	61	61

Investigations into anion effect were also conducted and it was observed that groups containing the octyl sulfate anion appeared more readily degradable. Further alterations of the alkylimidazolium cation were achieved by incorporating various oxygenated moieties such as polyethylene glycol. It was demonstrated that long chains could be attached to the cation core without increasing the antimicrobial toxicity by incorporated ether linkages.

Comprehensive data sets, have been reported, on both the cation and anion effects on biodegradation and toxicity of ILs [52,53,61,62,63].

The library of compounds produced also illustrates the potential for a rational design process in pharmaceuticals and surfactants with a requirement for environmental friendliness in compounds produced and a potential for “benign by design” [64].

The consequence of the tandem approach of biodegradation and toxicity screening manifested itself in the recent “hit” MRSA selective molecule synthesized by the Gathergood group as part of a larger library of ionic liquids. The hit molecule, shown previously in Figure 4, structure (**5**), was the cumulative effort of years of research into biodegradability of ILs. Previous work performed by the group demonstrated that the introduction of ester groups into long alkyl chains, using the imidazolium cation in the ILs, reduced toxicity and improved ecotoxicity of the compounds. From here, introduction of ether linkages were shown to further increase biodegradability. Conversely, it was demonstrated that pyridinium ILs containing ester groups and/or amide analogues exhibit a much higher resistance to biodegradation. The hit molecule exhibits clinically relevant levels of MRSA growth inhibition, see Table 2, ILs **5** (125 μM, IC_95_). The eventual aim of the research project is to produce an MRSA selective antimicrobial with high selectivity that is readily biodegradable.

The potential of ILs as alternatives to “hard” antimicrobials may also be exploited. “Soft” antimicrobials are described as biologically active compounds that are readily degraded into nontoxic and benign chemicals *in vivo*. A series of QACs containing labile ester and amide spacer groups, and long lipophilic alkyl chains have been reported as soft antimicrobials. Amongst other relevant examples of soft antimicrobials, synthesized using green chemistry concepts, are the examples of bis-arginine surfactants that were shown to cause membrane damage and potassium leakage from a number of gram-positive bacteria [65].

## 15. Conclusions

Little is currently known about the potential health effects on populations exposed to trace amounts of antibiotics and how they could affect our ability to combat disease and live healthy lives. 

From the observations highlighted in this review and numerous studies performed in the field, it has been widely accepted that accumulation of pharmaceutical compounds in the environment can affect animal and plant life. Whether or not a chemical compound has a known effect in the environment, best practice is to avoid persistence. The long term effects cannot always be known until a protracted period of time has elapsed. For example, are persistent antibiotics directly linked to the increasing number of resistant bacterial strains? It is difficult to give a definite answer to that question without large scale sampling over a long period of time. Therefore, we propose it is better to adopt a precautionary approach.

Giving bacteria the opportunity outside (or inside) hospitals the possibility to develop resistance to new drugs counteracts the progress of creating “better” antibiotics, if their efficacy is allowed to be undermined.

Similarly, the recent outbreak in Europe in the summer of 2011 of Enterohaemorrhagic *Escherichia coli* (EHEC) which was determined to have originated from contaminated water sprayed on vegetables also brings forth the concern that resistant strains of bacteria in water sources could potentially be linked to persistent antibiotics in the environment.

With the ever increasing acceptance of green chemistry metrics as benchmarks in pharmaceutical processes, the potential for a new age of cleaner more environmentally acceptable pharmaceutical production and research is possible. Solvent selection, waste recycling and integration of biological reactions and enzymatic steps has been shown to be highly successful in replacing traditional organic methods of synthesis. By adopting lifecycle assessment and biodegradation/toxicity parameters into a drugs design program the potential for environmental persistence of antibiotics can be reduced. The technology and the datasets for predicting a drugs characteristics are only getting better as time goes on, it is whether there is the will or the initiative to employ such methods in research that will decide how persistence of antibiotics in the environment is dealt with, or ignored.
